# Graphene nanoribbon field-effect transistor at high bias

**DOI:** 10.1186/1556-276X-9-604

**Published:** 2014-11-06

**Authors:** Mahdiar Ghadiry, Razali Ismail, Mehdi Saeidmanesh, Mohsen Khaledian, Asrulnizam Abd Manaf

**Affiliations:** 1 Faculty of Electrical Engineering, Universiti Teknologi Malaysia, Skudai, Johor Darul Takzim 81310, Malaysia; 2 School of Electrical and Electronic Engineering, Univesiti Sains Malaysia, Pulau Pinang 14300, Malaysia

**Keywords:** Graphene, High bias, Current, Breakdown, Model, Fabrication

## Abstract

Combination of high-mean free path and scaling ability makes graphene nanoribbon (GNR) attractive for application of field-effect transistors and subject of intense research. Here, we study its behaviour at high bias near and after electrical breakdown. Theoretical modelling, Monte Carlo simulation, and experimental approaches are used to calculate net generation rate, ionization coefficient, current, and finally breakdown voltage (BV). It is seen that a typical GNR field-effect transistor's (GNRFET) breakdown voltage is in the range of 0.5 to 3 V for different channel lengths, and compared with silicon similar counterparts, it is less. Furthermore, the likely mechanism of breakdown is studied.

## Background

Scaling in CMOS technology has been the key action to improve the power and performance of field-effect transistors [1]. As a result, there is a continuous need for thinner and shorter channels to resolve problems such as short channel effects in modern transistors. However, this scaling trend could not continue for long with silicon as the channel material. Recently, graphene has been introduced as an alternative for silicon hoping that this trend could go on further. But the short channel and high-mean free path of graphene up to 400 nm [2] result in high ionization rate and breakdown at high biases. Therefore, it is important to study the breakdown in graphene-based transistors.

Breakdown current density in graphene has been reported number of times mostly to study their application in on-chip electrical interconnects using several experimental approaches. In [3], mechanically exfoliated graphene nanoribbons (GNRs) were found to display an impressive current-carrying capacity of more than 10^8^ A/cm^2^ for the widths down to 16 nm. In addition, breakdown voltage (BV) is estimated to be around 2.5 V for GNRs with widths of 22 nm. Chemical vapour deposition (CVD) was used by Lee et al. [4] to fabricate multilayer graphene sheets having an average thickness of 10 to 20 nm. They reported the breakdown current densities of up to 4 × 10^7^ A/cm^2^. In addition, graphene wires with widths of 1 and 10 μm and lengths from 2 to 1,000 μm have been fabricated, and the breakdown voltage is reported to be around 8 V. Epitaxial graphene developed on silicon carbide is studied in terms of breakdown current in [5]. They prepared Hall bar structures of different sizes (*W* =0.5 to 5 μm, *L* =8 to 25 μm) by e-beam lithography, and maximum current density, mobility, and charge carrier density are measured. It is reported that the graphene film breaks down at a critical current density of 4 to 6 mA/μm. In another work, the BV of GNR field-effect transistor (GNRFET) is reported to be in the range from 0.25 to 0.65 V for 50-nm GNR with widths from 3 to 6 nm [6]. They used analytical approach to calculate the breakdown voltage and ionization coefficient in double-gate GNRFET. In modelling, Gauss's law and Poisson's equation [6-8] were applied to derive surface potential equation and the lucky drift theory to calculate the ionization coefficient [6]. However, they did not take the effect of ionization coefficient into account for surface potential modelling, which makes their model inaccurate for GNR. In addition, they used Monte Carlo approach to simulate ionization coefficient, while we extend the approach to calculate net generation rate. The effect of carrier generation once used in graphene field-effect transistor in [9] is different with our work in two ways: first, we study the GNRFET, and second, in this paper, we measure and model the breakdown voltage and current at high bias near breakdown, while that paper only derives the current.

## Methods

### Breakdown of GNRFET

**Figure 1:**
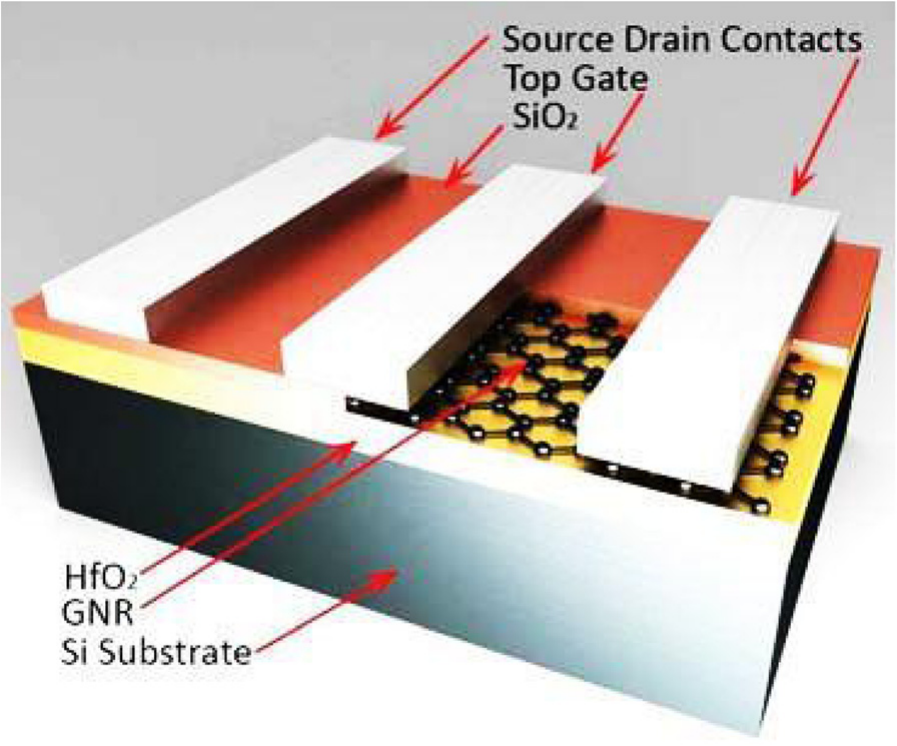
**Cross section of the device used for modelling and fabrication.** The dimensions of the layers are as follows: (from top) GNR thickness *t*_g_ =0.4 nm, oxide thickness *t*_ox_ =20 nm, contact thickness *t*_Au_ =100 nm, channel length *L* =100 nm, and channel width *W* =30 nm.

Figure 1 shows the device used for modelling and fabrication in this project. The silicon substrate serves as the back gate. Identification of graphene flakes is done using optical approach and Raman spectroscopy. Drain, source, and gate contacts are patterned by several steps of electron beam lithography followed by metal deposition, and a HfO_2_ layer is formed by low-temperature atomic layer deposition (ALD), which is used to form SiO_2_, too.

We start the modelling by electron and hole current density 1D continuity in graphene sheet channel yielding

(1) $$\frac{d\left(J_{p}-J_{n}\right)}{\mathrm{dy}}=2\alpha_{i}q\text{,}$$

where *α*_i_ is the electron generation rate due to ionization, *J*_p_ and *J*_n_ are current densities of holes and electrons, respectively, and *q* is the electron charge. The generation rate is normally ignored in silicon devices; however, according to [10], in graphene, this parameter is not ignorable. Integrating over Equation 1 results in

(2) $$I_{p}-I_{n}-2qW\int_{y}^{L}\alpha_{i}\mathrm{dy}=I\text{,}$$

where *I* is the total channel current and *I*_n_ and *I*_p_ are electron and hole currents, respectively. Using charge equation, *Q*_+_ − *Q*_−_ = ± *C*_*top*_(*V*_*gt*_ − *V*_*bg*_ − *φ*(*y*)), one can write

(3) $$C_{\mathrm{top}}W\left(V_{gt}-V_{bg}-\varphi ,\left(y\right)\right)\nu_{d}+2qW\int_{0}^{y}\alpha_{i}\mathrm{dy}=I$$

where $v_{d}=\frac{\mu E\left(y\right)}{1+E\left(y\right)/E_{c}}$ is the drift velocity, *μ* is mobility, *E*(*y*) is the electric filed, *E*_c_ is the critical electric field, *φ*(*y*) is surface potential, *V*_bg_ and *V*_gt_ are back- and top-gates, respectively, and *C*_top_ *= C*_q_*C*_ox_ / *C*_q_ + *C*_ox_, where *C*_ox_ and *C*_q_ are classic and quantum capacitances, respectively, of the gate given by *C*_ox_ *= ϵ*_ox_ / *t*_ox_ and *C*_q_ =2 *μF*/cm^2^, respectively, with *ϵ*_ox_ being oxide dielectric constant.

Therefore, drain current equation could be written by integrating from source (*y* =0) to position *y* along the channel over Equation 3. As a result, we have

(4) $$\begin{array}{ll} I= & \frac{E_{c}W}{E_{c}+\varphi \left(y\right)}\left(C_{\mathrm{ox}},\mu ,\left(V_{gt}-V_{bg}-\frac{\varphi \left(y\right)}{2}\right),\varphi ,\left(y\right)\right)\\ & \left(+y+\frac{\varphi \left(y\right)\alpha_{i}}{E_{c}}\int_{0}^{y}\left(y^{\prime }+\frac{V\left(y^{\prime }\right)}{E_{c}}\right)dy^{\prime }\right) \end{array}$$

from which the surface potential is written as

(5) $$\varphi \left(y\right)=\frac{\alpha_{i}y^{2}E_{c}W-2\alpha_{i}W\int_{0}^{y}V\left(y^{\prime }\right)dy^{\prime }-2E_{c}I}{2I-E_{c}WC_{\mathrm{ox}}\mu {\left(V_{gt}-V_{bg}-\frac{\varphi \left(y\right)}{2}-2\alpha_{i}Wy\right)}^{\prime }}$$

where total current *I* according to [11] could be replaced by $I_{d}=WqV_{ds}{\left(\int_{0}^{L}\frac{E\left(y\right)}{n\left(y\right)\upsilon_{d}}\right)}^{-1}$, where *n*(*y*) is the carrier concentration of GNR and *V*_ds_ is the drain-source voltage.

Therefore, by using *φ*(*L*_d_) = *V*_sat_, where *V*_sat_ is the drain saturation voltage, one can write the equation of the saturation region length (*L*_d_) as

(6) $$L_{d}=\frac{V_{sat}\left(2E_{c}WC_{\mathrm{ox}}\mu \left(V_{gt}-V_{bg}-\frac{\varphi \left(y\right)}{2}\right)-2I-2\alpha_{i}\int_{0}^{L_{d}}V\left(y^{\prime }\right)dy^{\prime }-2E_{c}I\right)}{-\alpha_{i}W\left(2V_{sat}+L_{d}E_{c}\right)}$$

Finally, applying avalanche breakdown condition [12], the breakdown voltage can be numerically calculated from $\int_{0}^{L_{d}}\alpha dx=1\text{,}$ where *α* is the ionization coefficient calculated by Monte Carlo simulation.

### Monte Carlo simulation

Two scattering mechanisms of (i) elastic scattering by acoustic phonons, which is the dominant scattering mechanism at low carrier energies in GNR [13], and (ii) inelastic scattering via emitting an optical phonon of energy *ℏω*_op_, which is the dominant scattering mechanism at high energies [13], are considered to be influential on the carrier trajectory. They are characterized by the associated individual mean free paths *λ*_e_ and *λ*_ie_ , respectively. The impact ionization takes place immediately after the carrier builds the kinetic energy equal to the ionization threshold *E*_t_. The impact ionization coefficient is defined as *α* =1 / *Z*[14], where *Z* is the average distance travelled by the carrier in the field direction prior to the ionization. We use a self-scattering approach, which introduces a fictitious forward scattering in order to eliminate solving integral equations in every Monte Carlo step. The self-scattering rate *R*_ss_ is calculated from *R*_ss_ = *υ*_*g*_ / *λ*, where *λ* =1 / *λ*_*m*_ +1 / *λ*_*ie*_, where *λ*_m_ and *λ*_ie_ are the momentum and energy mean free path. Free flight time (*dt*) is calculated from *dt* = −1 / *R*_ss_ ln(*r*), where *r* is a random number between 0 and 1. The wavevector and position vector ***X*** are given by *d* ***k*** = *q* ***F*** *dt* / *ℏ* and $dk=qFdt\;/\;\hbar \;\mathrm{and}\;X=k\frac{\hbar dt}{m}+qFdt^{2}/2m$, respectively. The kinetic energy of GNR is calculated from *E*_k_ = *ℏ*^2^ ***k***^2^ / *m* ∗, where m* is the effective mass of GNR. If *E*_k_ = *ℏω*_op_, then *R*_ie_ is assigned a non-zero value. For elastic or inelastic scatterings, the orientation of ***k*** is changed, while for self-scatterings, the ***k*** vector remains unchanged. Finally, the net generation rate due to impact ionization could be calculated from *α* = *n*_*2*D_(*L* − *x*) / *t*, where *n*_2D_ is the two-dimensional carrier concentration.

## Results and discussion

**Figure 2:**
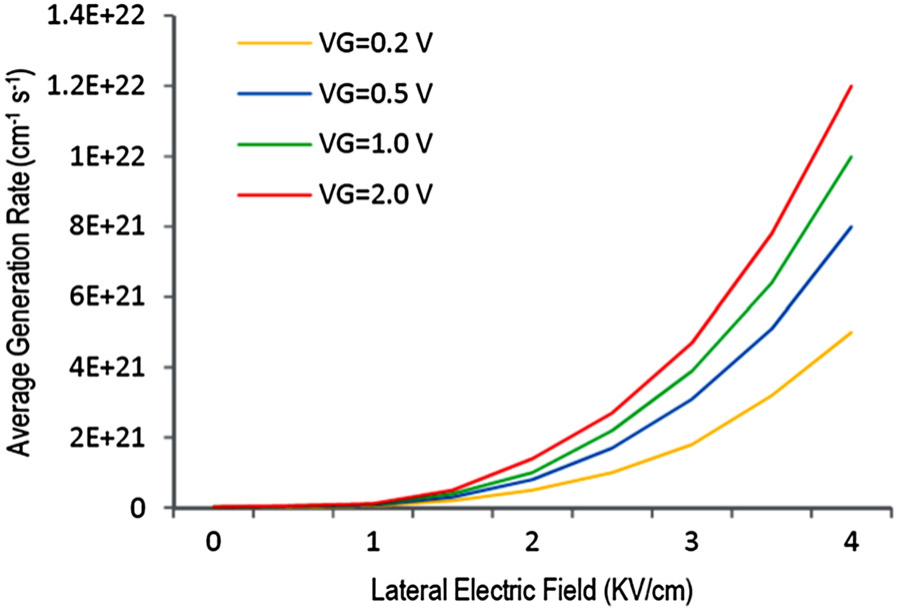
**Carrier net generation rate *α***_**i**_** as a function of lateral electric field *E*** **(*y*)** ***.***

**Figure 3:**
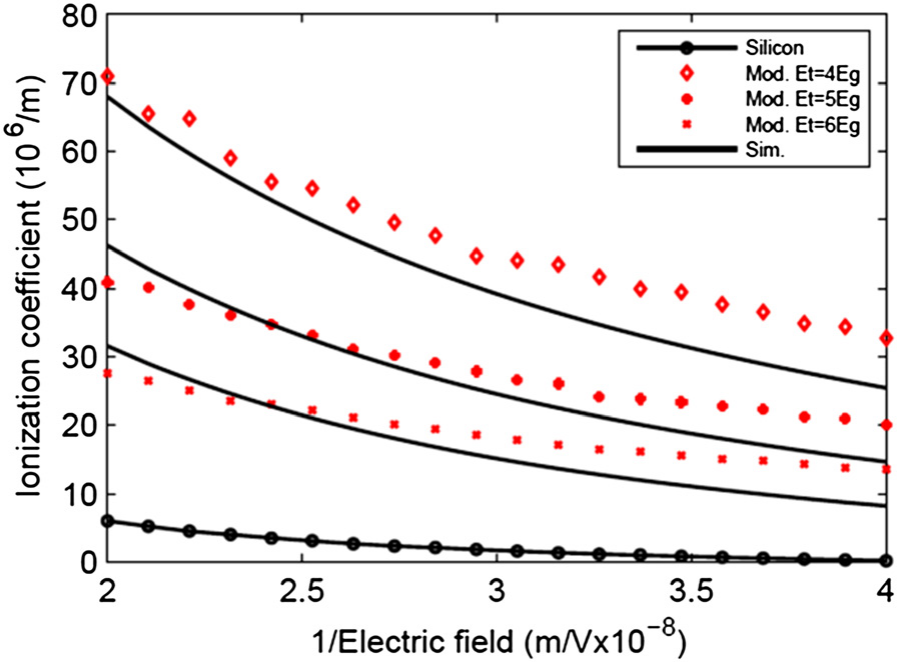
**Ionization coefficient** ***α*** **as a function of reciprocal electric field and ionization threshold energy** ***E***_**t**_**.**

In order to calculate BV, firstly, we need to know the values of net generation rate *α*_i_ and ionization coefficient *α*, which are simulated using the Monte Carlo approach presented. The values of *α*_i_ versus lateral electric field at different gate voltages are shown in Figure 2. In addition, in Figure 3, the ionization coefficient of GNR at different ionization threshold energies is depicted. Comparing silicon (extracted from [12,15,16]) with GNR shows that the ionization event in GNR is much more than that of silicon, which is attributed to its high-mean free path resulting in early velocity saturation of carriers. The solid lines in these two figures show the simulated data using Monte Carlo, and the red dots are the modelling data from [6]. There is discrepancy between the two approaches. In the modelling, the energy and momentum mean free time (*τ*_E_ and *τ*_m_) are used to calculate the probability of energy and momentum relaxing collisions. For simplicity, it has been assumed that drift velocity is not a function of energy. In addition, the energy mean free time is calculated from $\tau_{E}\;=\frac{E\tau_{m}}{\hbar w_{\mathrm{op}}}$ since it has been assumed that the dominant scattering mechanism in graphene is phonon scattering ignoring acoustic phonon scattering mechanism, while in the Monte Carlo approach, it has been taken into account and drift velocity is a function of energy.

**Figure 4:**
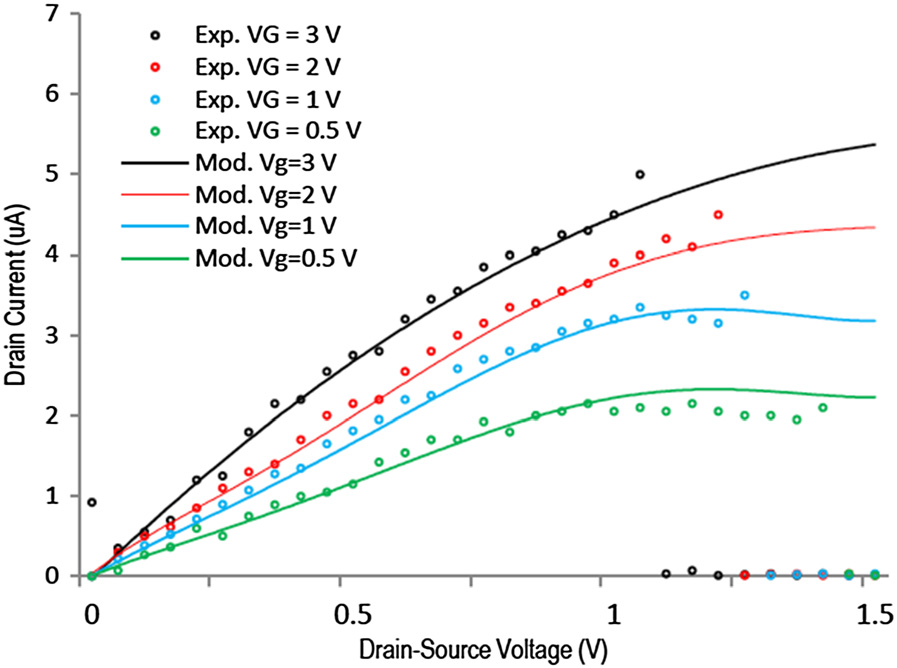
**Current of a 100-nm device at different gate voltages.** In the modelling of the current, the breakdown effect is not considered, while sudden current increase is the sign of breakdown in experimental data.

**Figure 5:**
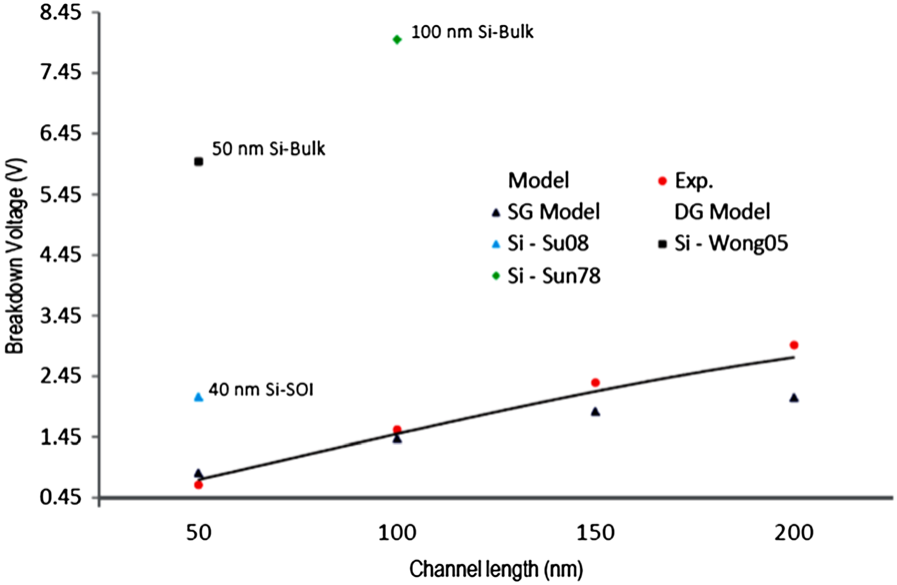
**Breakdown voltage of different devices in terms of channel length at certain gate voltage of 0.25 V.** The solid line is the theoretical data, and the dots are the experimental data.

**Figure 6:**
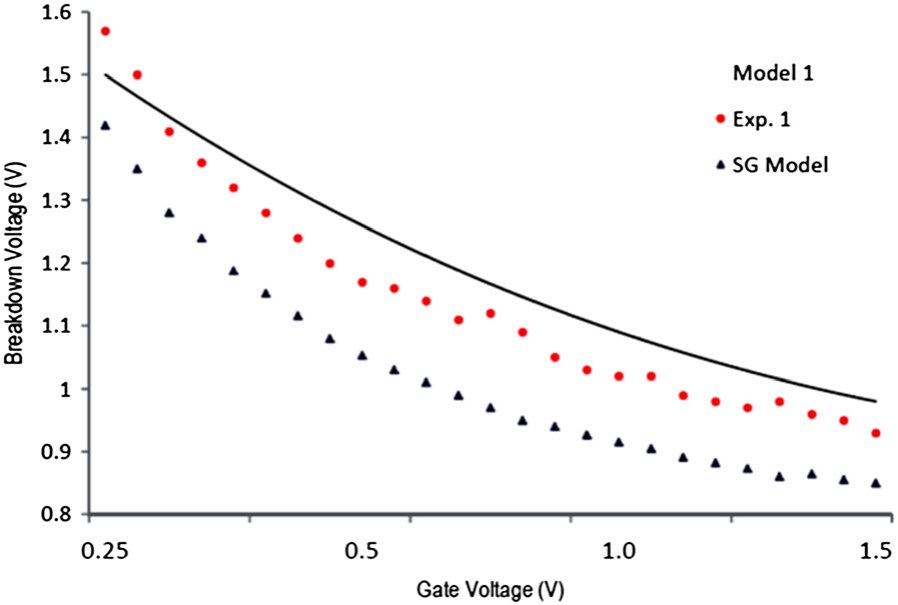
**Breakdown voltage of a 100-nm device at different gate voltages ranging from 0.25 to 1.5 V.** Solid line is the theoretical data, red dots are the experimental data, and blue triangles show the data from the proposed model for single-gate GNRFET [6].

Next, we measure the drain current and compare with the modelling data in Figure 4. The markers show the experimental data, and the solid lines show the modelling data at different drain and gate voltages. There is a sudden current stop showing breakdown, which points to Joule heating as the likely mechanism of breakdown and a rise in current before current stop, which we associated to three phenomena of self-amplifying excess carrier generation caused by ionization, substrate contribution in current at high temperature, and generation of more conducting channels. Using current data at different conditions, the BV for different devices is obtained and shown in Figures 5 and 6. In Figure 5, four devices are fabricated and used with 50-, 100-, 150-, and 200-nm channel lengths at 0.4-V gate voltage. It is found that longer channel results in higher breakdown voltage ranging from almost 0.45 V for 50-nm to 1.65 V for 200-nm devices. The experimental data shown by red dots agree with the modelling data shown by solid lines. The experimental data from similar silicon-based devices are also shown in this figure. It is seen that GNR breaks down at lower voltages, which is the sign of ballistic carrier transport in graphene, resulting in hot carriers. In addition, in Figure 6, the gate voltage is changed from 0.2 to 1.4 V to examine its effect on the breakdown voltage. It is seen that the breakdown voltage could be 1.4 V for a 100-nm device at 0.2-V gate voltage. In addition, the theoretical data from [5] for breakdown of a typical single-gate device is shown by blue triangles. In theory, the effect of substrate and heating process has not been considered which we think is the reason for the small discrepancy between the theoretical and experimental data.

## Conclusions

The breakdown of graphene nanoribbon transistors was studied experimentally and theoretically in this report. Monte Carlo simulation was employed to simulate ionization rate and net generation rate. Then, the current is modelled and finally the breakdown voltage. In addition, we fabricated four devices, measured the breakdown voltage and current, and compared the voltage and current with those of the modelling data. A sudden rise and then a sudden stop were seen in the current profile which we associated with excess carrier generation and Joule heating, respectively. The breakdown of 50- to 200-nm devices was reported to be in the range of 0.5 to 3 V, which is less than that of counterpart silicon devices.

## Competing interests

The authors declare that they have no competing interests.

## Authors’ contributions

MG carried out the modelling part. RI made a major revision of the paper and led the team. MS, MK, and MG contributed in the fabrication and measurement, and AAM did the Monte Carlo simulations. All authors read and approved the final manuscript.
